# Identifying components for programmatic latent tuberculosis infection control in the European Union

**DOI:** 10.2807/1560-7917.ES.2016.21.34.30325

**Published:** 2016-08-25

**Authors:** Andreas Sandgren, Jannigje M Vonk Noordegraaf-Schouten, Anouk M Oordt-Speets, Gerarda B van Kessel, Sake J de Vlas, Marieke J van der Werf

**Affiliations:** 1 European Centre for Disease Prevention and Control (ECDC), Stockholm, Sweden; 2 Pallas health research and consultancy BV, Rotterdam, the Netherlands; 3 Department of Public Health, Erasmus MC, University Medical Center Rotterdam, Rotterdam, the Netherlands

**Keywords:** infectious disease, Mycobacterium tuberculosis, latent tuberculosis infection, Prevention and control, public health, health policy

## Abstract

Individuals with latent tuberculosis infection (LTBI) are the reservoir of *Mycobacterium tuberculosis* in a population and as long as this reservoir exists, elimination of tuberculosis (TB) will not be feasible. In 2013, the European Centre for Disease Prevention and Control (ECDC) started an assessment of benefits and risks of introducing programmatic LTBI control, with the aim of providing guidance on how to incorporate LTBI control into national TB strategies in European Union/European Economic Area (EU/EEA) Member States and candidate countries. In a first step, experts from the Member States, candidate countries, and international and national organisations were consulted on the components of programmatic LTBI control that should be considered and evaluated in literature reviews, mathematical models and cost-effectiveness studies. This was done through a questionnaire and two interactive discussion rounds. The main components identified were identification and targeting of risk groups, determinants of LTBI and progression to active TB, optimal diagnostic tests for LTBI, effective preventive treatment regimens, and to explore the potential for combining LTBI control with other health programmes. Political commitment, a solid healthcare infrastructure, and favourable economic situation in specific countries were identified as essential to facilitate the implementation of programmatic LTBI control.

## Introduction

### Control of latent tuberculosis infection

The epidemiological situation of tuberculosis (TB) in the European Union/European Economic Area (EU/EEA) is heterogeneous. Substantial differences are seen in the TB notification rates and different countries face different challenges such as TB among migrants [1]. In 2013, 18 EU/EEA countries had less than 10 TB cases per 100,000 population [1] and are considered to have entered the TB elimination phase [2]. To reach TB elimination, a comprehensive package of interventions is required that includes addressing latent tuberculosis infection (LTBI) [3]. Individuals with LTBI represent a source from which active TB disease arises [4] and LTBI control is therefore an important condition for TB elimination. Detection of individuals with LTBI and provision of preventive treatment to these cases are key principles of LTBI control. Some countries have implemented LTBI interventions, for example in the United Kingdom (UK), individuals infected with human immunodeficiency virus (HIV) are tested for LTBI, and in the Netherlands, specific high-risk groups are targeted for LTBI screening [5,6]. As more countries are reaching the elimination phase, it is relevant to consider implementing a programmatic approach to LTBI control in the EU/EEA and candidate countries, which implies a national level comprehensive and systematic strategy.

### Aim and scope of the ECDC project

The European Centre for Disease Prevention and Control (ECDC) has embarked on a project to provide EU/EEA Member States and candidate countries with scientific advice and guidance on programmatic LTBI control. In 2013, ECDC therefore initiated a comprehensive assessment of the potential benefits and risks of introducing programmatic LTBI control in national TB prevention and control strategies. The assessment is being carried out by a consortium consisting of Pallas health research and consultancy and the Department of Public Health at Erasmus Medical Center, both located in Rotterdam, the Netherlands. The goal of this assessment is to develop guidance that provides options for programmatic LTBI control. The assessment includes the following activities:

1. Inventory of expert opinions on components of LTBI control to consider in the assessment, collected through a questionnaire and two interactive rounds during a workshop;

2. Systematic literature reviews on scientific evidence for relevant components of LTBI control;

3. Mathematical modelling and cost-effectiveness studies on LTBI control;

4. Expert panel meeting to discuss the results of activities 2 and 3;

5. Strategy synthesis and guidance development with the options for introducing programmatic LTBI control in the EU/EEA.

Here we provide an overview of the inventory of expert opinions (activity 1).

## Methods for inventory of expert opinions

The experts’ opinions were collected in a modified Delphi approach with three rounds (round 1: questionnaire, round 2: Treasure Hunt and round 3: Idea Factory). The objectives of this inventory were: (i) to define the components of programmatic LTBI control in the EU/EEA to be considered and evaluated during the assessment and (ii) to develop research questions on each component of programmatic LTBI control for the systematic reviews.

The questionnaire round was held in the months preceding the workshop meeting to collect the opinions and visions on LTBI control of 27 experts from EU/EEA Member States and candidate countries as well as six additional stakeholders in the field of TB (see acknowledgements for the list of participants). Country experts were nominated by the ECDC advisory forum (one expert per country) and stakeholder experts were selected by ECDC. The questionnaire collected background information about the expert, the TB situation in their country, an appraisal of the relevance, importance, efficacy, cost-effectiveness, acceptability, and feasibility of possible components of LTBI control, and the expected developments in TB epidemiology, control, and interventions. It also included questions about the best approaches for LTBI control in the expert’s country and in the EU/EEA as a whole. The outcomes of this questionnaire gave insight into the aspects the experts agreed and disagreed upon. These were used to define the components of programmatic LTBI control to be further evaluated in the second and third round of the Delphi process.

The questionnaire round was followed by a workshop meeting on 19–20 September 2013. During the workshop, the participants received a summary of the questionnaire results, which they further discussed using the methods ‘Treasure Hunt’ (round 2) and ‘Idea Factory’ [7] (round 3). The methods and themes for discussion were adapted using the results of the questionnaire round. Both interactive methods allowed the participants to further identify and refine relevant components of LTBI control and to assess what aspects were key for the successful implementation of the components.

Treasure Hunt is a method that allows for an efficient and intense exchange of ideas. It comprises of a number of interactive rounds on central themes or questions which are discussed in small groups. In our project, six questions were discussed, aiming at: (i) the difference between TB and LTBI control, (ii) interventions with impact on LTBI incidence, (iii) risk groups, (iv) country-specific factors for LTBI control, (v) new diagnostics for LTBI and (vi) current developments in the EU/EEA regarding TB/LTBI. The Idea Factory is an interactive process in which the experts, divided into small teams, are asked to develop proposals regarding several themes defined beforehand. The themes for round 3 were adapted to the outcomes of round 2. The process is shaped as a competition where proposals are evaluated by a review team. In developing the proposals, the participants were asked to elaborate on the following aspects: specific conditions needed for successful implementation of interventions in LTBI control programmes, circumstances that should be taken into account during the implementation of the intervention, and who should have the lead. In a concluding session, the experts were asked to suggest research questions relevant for the next steps in the assessment, in particular for the systematic reviews to be performed.

## Inventory of expert opinions

### Questionnaire

In total 23 of the 27 experts filled out the questionnaire. The appraisal considered contact tracing a very important intervention to control LTBI. Chemoprophylaxis (for individuals at risk of TB infection) and preventive therapy (for individuals with LTBI) were seen as relevant interventions by most experts, but not as feasible interventions. Screening programmes to detect LTBI among high-risk groups were also frequently seen as a relevant and important intervention, but were considered not always feasible. Vaccines against TB infection were valued as an acceptable intervention, but not as a feasible intervention. The results of the questionnaire showed that LTBI control should mainly focus on high risk groups such as (but not limited to) HIV-infected individuals, healthcare workers and immunocompromised patients, but not on travellers to countries with a high TB incidence or people who abuse alcohol. During the meeting, TB contacts and migrants or refugees were also suggested as a target group for LTBI control. Overall, more than 90% of the experts thought that programmatic LTBI control in their country and in the EU/EEA would be relevant, important and effective. LTBI screening (in high-risk groups) was considered the best and most complete intervention to control LTBI in their country and in the EU/EEA.

### Treasure Hunt

During the Treasure Hunt, six questions were discussed by the experts:

####  1. What is the difference between TB and LTBI control?

The experts indicated that TB and LTBI control are closely related in terms of case finding, treatment, treatment-related side-effects, risk groups, stigma and the goal of the control programme (i.e. decreasing TB incidence). However, there are also important differences between the two: active TB disease is infectious, the methods of diagnosis and treatment of TB and LTBI are distinct, the ethics regarding treatment (treating ill persons with TB vs ‘not ill’ persons with LTBI) and the acceptability of possible side effects of TB or LTBI treatment differ. As a result, the perception and understanding of infection vs disease among policymakers and healthcare workers are also different. The participants emphasised that they expected that evidence-based data for LTBI control strategies are currently not widely available.

####  2. What do you consider the most important interventions with impact on LTBI incidence?

The interventions that the participants considered to have the largest impact on LTBI in Europe within the next 10 years were contact tracing, LTBI screening and preventive therapy for LTBI, especially in risk groups. Furthermore, a need for developing better tests for LTBI diagnosis was identified, as was a need for prognostic tools that predict the chance of active TB developing in individuals infected with *Mycobacterium tuberculosis*. Also, better insight into the prevalence and determinants of progression to TB disease was considered desirable.

####  3. What are the risk groups that should be prioritised for LTBI control interventions?

According to the experts, besides TB contacts and immunocompromised patients, HIV patients as well as migrants and refugees should have the highest priority in programmatic LTBI control. The risk group consisting of travellers to countries with high TB incidence should have the least priority. LTBI interventions should be country-specific, however.

####  4. What are country-specific factors for LTBI control that should be taken into consideration?

Although the meeting participants considered it possible to identify LTBI control interventions that could be implemented in the EU/EEA in general, there are country-specific factors that should be taken into account to successfully implement these interventions, such as the epidemiological situation (e.g. overall TB incidence, incidence of multidrug-resistant TB (MDR-TB)), healthcare structure and infrastructure (e.g. health priorities, medical partnerships, local feasibility), cultural aspects (e.g. acceptance by clinicians of LTBI treatment as a useful intervention ) and available resources.

####  5. What are the arguments for investment in new diagnostics for LTBI?

The expert groups were asked to provide arguments both for and against the proposition that extra attention and financial resources should be invested in new diagnostics for LTBI. All expert groups provided arguments in favour of the proposition that there is a need to develop better diagnostic tests and prognostic tools. The most important arguments mentioned were: to save costs, to have a better tool for LTBI diagnosis and to be able to gain better insight in LTBI such as the prevalence and determinants of progression to TB disease. Half of the expert groups also provided arguments against the proposition. These were that money and focus could better be used for other investigations such as studies on MDR-TB and the development of a vaccine.

####  6. What are the key future developments in the EU/EEA regarding TB/LTBI?

When considering LTBI prevention strategies, it is important to take future developments in Europe into account, such as changes in migration patterns, incidence of MDR-TB and of HIV/TB co-infection, and waning TB expertise among healthcare professionals as TB becomes less common.

### Idea Factory

During the Idea Factory, proposals for the implementation of the following seven themes and an open category regarding programmatic LTBI control in the EU/EEA were developed by the participants: contact tracing, chemoprophylaxis, preventive therapy, screening, education and information, programmatic LTBI control in the EU/EEA and integration of latent TB control in other healthcare interventions. The main proposals are summarised in Box 1. An important condition for successful implementation mentioned in most of the proposals was to ensure political will and commitment.

Box 1 **Main proposals developed during the Idea Factory for the implementation of programmatic control of latent tuberculosis infection^a^**

To develop a systematic approach to implement contract tracing in programmatic LTBI control;
To develop monitoring and evaluation systems for contact tracing, for LTBI cases and for outcome of preventive treatment;
To use social networks to improve contact tracing;
To identify the target groups for chemoprophylaxis and preventive treatment;
To collect more evidence on the compliance with and outcome of LTBI treatment in the different target groups;
To ensure education and training for all levels of society, including specific groups such as policy makers, healthcare workers and community workers;
To develop methods and content for the information and education strategy and take into consideration the specifities of the target groups;
To integrate LTBI control in healthcare programmes for other diseases;
To invest in research and development of better drugs;
To provide support and technical assistance for development and implementation of guidelines;
To develop a decision-making support tool for LTBI control.

^a^ Experts elaborated on conditions needed for successful implementation of interventions in LTBI control programmes, circumstances that should be taken into account during the implementation and who should have the lead (not reported in this box).

## Research questions for systematic reviews

Based on the outcomes of the questionnaire and the two rounds of interactive discussion, the participants developed topics for research questions to be addressed in the systematic literature reviews. The main themes of the research questions on LTBI control were: identification of the most important LTBI risk groups, prevalence of LTBI in different risk groups and the general population, risk of active TB over time after infection, risk of TB after exposure to an infectious index case with or without preventive therapy, risk of developing TB related to the country of origin when migrating to a low incidence area, current most optimal diagnostic test or combination of diagnostic tests for diagnosing LTBI, efficacy of and current most optimal LTBI preventive treatment regimens in different risk groups, major and minor adverse events related to LTBI preventive treatment, adherence to LTBI preventive treatment in different risk groups, effective interventions to improve LTBI treatment adherence, access to risk groups for screening and treatment, impact of combining LTBI screening with other health programmes and increasing awareness and knowledge of LTBI.

## Summary of main components for the assessment

According to the consulted experts, there are a number of issues that ECDC and the EU/EEA Member States need to assess and get a more comprehensive perspective about before deciding to include programmatic LTBI control in the EU/EEA.

Firstly, the prevalence of LTBI in specific risk groups and the respective risk of progression to active TB disease should be assessed. This includes assessing factors and determinants that influence the prevalence of LTBI (in particular changing migration patterns), and the risk of developing active TB over time in infected persons, with or without chemoprophylaxis or preventive treatment.

Regarding the diagnosis of LTBI, experts considered it important to identify the most reliable tests with the highest yield in different epidemiological settings and populations (e.g. immunocompromised patients, HIV patients, children, migrants and close contacts of TB patients). Also, the best strategy for LTBI and TB screening and case finding should be assessed, as well as the potential for combining this with other health programmes. An assessment of the legislation and potential changes needed to implement screening programmes was also suggested.

Furthermore, assessing the best preventive treatment regimens for LTBI in different situations and in different target groups, considering efficacy and adverse effects will be important. The effectiveness of different interventions to improve LTBI treatment uptake and adherence should be assessed, such as directly observed treatment (DOT) and incentives, including making LTBI diagnosis and treatment free of charge. It is likely that programmatic LTBI control will focus on risk groups for TB. The experts therefore suggested that questions on how to best target risk groups and improve their access to LTBI screening and treatment should be addressed.

The experts further suggested to look at interventions based on information and education to increase awareness and knowledge of LTBI and TB, targeting different groups such as policymakers, healthcare workers, medical students, community workers, risk groups and the population as a whole. The assessment should consider what the content of the education and information strategy should be, what the most effective methods for distributing information are and whether social networks can be used. Furthermore, the existence of guidelines and standardised methods for a programmatic LTBI control approach was highlighted as important, as well as the processes for evaluating the implementation of LTBI treatment programmes.

Finally, political will and commitment, the healthcare infrastructure, the economic situation, and other country-specific conditions and circumstances within EU/EEA Member States will have an impact on the implementation of programmatic LTBI control.

## Concurrent developments

In the concluding discussions of the workshop, experts emphasised the importance of harmonising and coordinating the assessment undertaken by ECDC with other activities in the area of TB elimination and LTBI control e.g. by the World Health Organization (WHO) and the European Respiratory Society. In keeping with this conclusion, when WHO embarked on developing a guideline on the management of LTBI in 2013, it did so in bilateral collaboration with ECDC and similarly, the ECDC project on programmatic LTBI control has been undertaken in collaboration with WHO. Since 2014, WHO and ECDC have collaborated and shared the evidence base on LTBI management and control which was collected through a series of systematic reviews. This information was used in WHOs 2015 guidelines on management on LTBI [8] and will be used by ECDC for further assessment (including mathematical modelling and cost-effectiveness analyses) and the development of guidance for programmatic LTBI control tailored to the EU/EEA.

## Concluding remarks

The workshop helped facilitate an exchange of insights between experts on different areas of LTBI control in Europe. It also created a platform for raising support for programmatic LTBI control that should increase the likelihood of cooperation and implementation during later phases of the process. Key areas that need further attention in the assessment of the potential benefits and risks of introducing programmatic LTBI control in the TB prevention and control strategy of the EU/EEA were identified and agreed upon. The input of the experts during the putting together of the inventory was not exhaustive, however, and the assessment that followed this process took into consideration additional relevant components and aspects, in collaboration with WHO.

Since the development of the inventory, the assessment has continued and a series of systematic literature reviews has been performed [9-14]. The next step will be to conduct mathematical modelling and cost-effectiveness studies. This work will contribute towards a guidance document that elaborates on the available options when considering programmatic LTBI control in the EU/EEA.
